# Postpartum vertebral artery dissection: case report and review of the literature

**DOI:** 10.1186/s12959-020-00243-w

**Published:** 2020-10-29

**Authors:** Nicholas T. Manasewitsch, Ahmed A. Hanfy, Bryce D. Beutler, Daniel Antwi-Amoabeng, Moutaz Taha, Mohamed Elnaggar, Gurpreet S. Chahal

**Affiliations:** Department of Internal Medicine, University of Nevada, Reno School of Medicine, 1155 Mill Street, W-11, Reno, NV 89052 USA

**Keywords:** Vertebral artery dissection, Postpartum, Reactive thrombocytosis, Pregnancy, Preeclampsia, Hypertensive disorders of pregnancy

## Abstract

**Background:**

Hypertensive disorders of pregnancy are associated with vascular complications, including ischemic stroke and cervical artery dissection. Vertebral artery dissection (VAD), however, is rare. We describe a 31-year-old female who presented with vertigo, nausea, and vomiting and was found to have a VAD. In addition, we discuss the presentation, differential diagnosis, and pathogenesis of this uncommon but clinically significant vascular event and summarize other cases of vertebral artery dissection described in the medical literature.

**Case presentation:**

A 31-year-old Hispanic woman presented 10 days postpartum with a one-day history of vertigo, nausea, vomiting, and frontal headache. The patient’s pregnancy course had been complicated by preeclampsia, chorioamnionitis, and iron-deficiency anemia, and her delivery was complicated by acute hemorrhage. Physical examination was significant for left leg ataxia. Laboratory studies showed marked thrombocytosis. Emergent computed tomography (CT) scan of the head was obtained and revealed a left cerebellar ischemic large vessel stroke. Subsequent CT angiography of the head and neck showed a left VAD. Based on correlation of the clinical history and laboratory and imaging findings, a diagnosis of vertebral artery dissection secondary to reactive (secondary) thrombocytosis from overlapping iron-deficiency anemia and acute hemorrhage was established. The patient was started on a heparin infusion and experienced significant improvement after a four-day hospitalization.

**Conclusion:**

VAD is a rare but important cause of neurologic symptoms in the postpartum period and should be considered in the differential diagnosis for women who present with headache and/or vertigo. Women aged 30 years or older and those with a history of a hypertensive disorder of pregnancy are at particularly high risk. Prompt diagnosis and management of VAD is essential to ensure favorable outcomes.

## Introduction

Hypertensive disorders of pregnancy (HDP) are associated with vascular events, including stroke (Liffert et al) and cervical artery dissection [1]. However, vertebral artery dissection (VAD) is rare [2, 3]. We describe a 31-year-old woman with a history of preeclampsia who presented 10 days postpartum with an ischemic posterior cerebellar stroke secondary to VAD. In addition, we summarize other cases of VAD in pregnancy. To our knowledge, this is the first report of postpartum VAD due to reactive thrombocytosis secondary to overlapping iron-deficiency anemia and acute hemorrhage.

## Case presentation

A 31-year-old Hispanic woman presented 10 days postpartum with a one-day history of vertigo, nausea, vomiting, and frontal headache. The patient’s pregnancy course had been complicated by preeclampsia without severe features, chorioamnionitis, and iron-deficiency anemia that was present before delivery. The patient underwent a primary low-transverse cesarean section at 40 weeks and 0 days that was complicated by an estimated 600 mL blood loss. The patient experienced persistent vaginal bleeding following delivery, but her postpartum course was otherwise uncomplicated. She was found to have a hemoglobin of 5.8 g/dL on postpartum day 3 and was transfused with one unit of packed red blood cells which increased her hemoglobin to 8.1 g/dL. The patient was discharged in stable condition later that day.

One day prior to the current presentation, the patient reported intermittent vertigo, gait ataxia, nausea, and bilious emesis. She subsequently developed a mild frontal headache. In addition, there had been persistent vaginal bleeding since her cesarean section. The patient denied fevers, chills, dyspnea, diplopia, syncope, falls, focal neurologic deficits, sensory loss, and photophobia.

The patient’s past medical history was unremarkable aside from a remote induced abortion. Appropriate prenatal care had been provided during pregnancy. The patient had a 16 pack-year smoking history, but she had quit all tobacco products upon learning that she was pregnant. She also endorsed occasional alcohol use prior to her pregnancy. There was no recreational drug use or significant family medical history.

Vital signs were within normal limits upon presentation. Physical examination revealed left leg ataxia; National Institutes of Health Stroke Scale (NIHSS) score was 1. Laboratory studies (reference ranges in parentheses) revealed platelet count: 1,003,000 /μL (164,000-446,000/μL), white blood cell count: 22,300 /μL (4800–10,8000 K/μL), hemoglobin: 11 g/dL (12–16 g/dL), mean corpuscular volume: 75 fL (81.4–97.8 fL), red blood cell distribution width – standard deviation: 52.1 fL (36–50 fL), serum iron: 22 μg/dL (40–170 μg/dL), total iron binding capacity: 683 μg/dL (250–450 μg/dL), percent iron saturation: 3% (15–55%), erythrocyte sedimentation rate: 39 mm/hr. (< 20 mm/hr), and C-reactive protein: 5.84 mg/dL (< 0.75 mg/dL). Urinalysis revealed cloudy urine, moderate occult blood, moderate leukocyte esterase, 20–50 white blood cells per high power field, and few bacteria. Peripheral blood smear showed anisocytosis and macrocytosis.

**Fig. 1:**
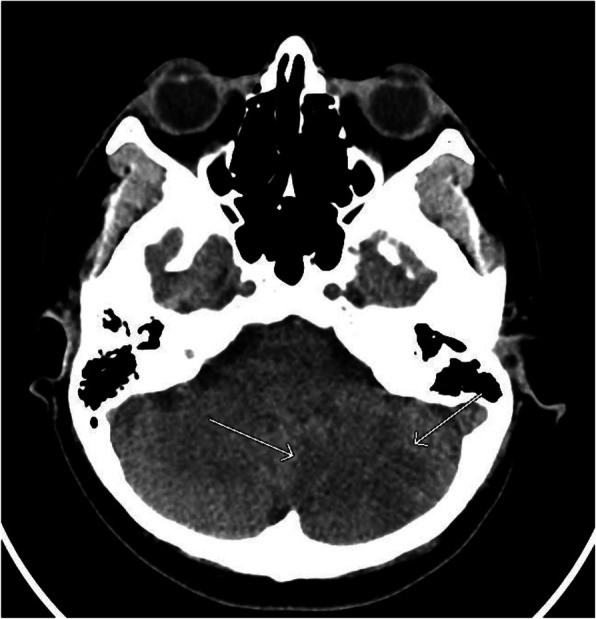
Non-contrast CT scan of the head demonstrates a probable acute or subacute infarction in the left cerebellar hemisphere

**Fig. 2:**
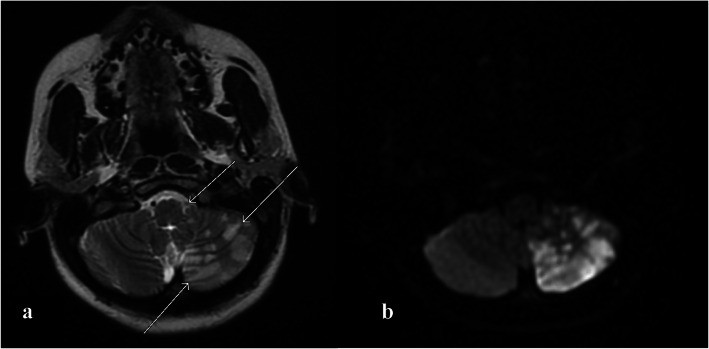
**a**. T2-weighted MRI of the brain demonstrates an extensive acute infarction of the left cerebellar hemisphere in the PICA territory and a left vertebral artery occlusion. **b**. Diffusion weighted MRI of the brain further confirms the extensive acute infarction of the left cerebellar hemisphere

**Fig. 3:**
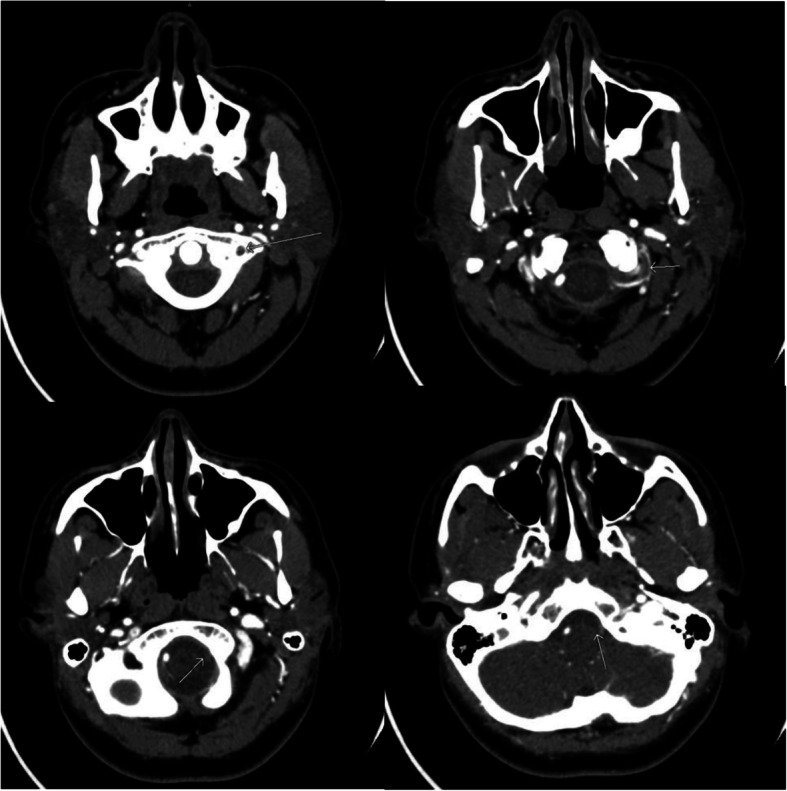
CT angiography of the head and neck demonstrates a left vertebral artery dissection originating at the C4 vertebral body level extending into the intracranial vertebral artery and involving the left posterior inferior cerebellar artery (PICA)

Computed tomography (CT) scan of the head without contrast showed patchy hypoattenuation involving the left cerebellar hemispheres suspicious for acute or subacute infarct (Fig. 1). Subsequent magnetic resonance imaging (MRI) of the brain without contrast showed an extensive acute infarction of the left cerebellar hemisphere in the territory of the posterior inferior cerebellar artery (PICA) as well as a left vertebral artery occlusion (Fig. 2). CT angiography of the head and neck showed a left vertebral artery dissection with significant flow limitation originating at the C4 vertebral body level extending into the intracranial vertebral artery and involving the left PICA (Fig. 3).

The patient was transferred to the intensive care unit and started on a heparin infusion. An extensive hypercoagulability workup was initiated; this included evaluation for mutations involving BCR-ABL, protein C, protein S, antithrombin III, factor V Leiden, prothrombin, anti-cardiolipin, beta-2-glycoprotein, MPL codon 515, CALR Exon 9 mutation, and V617F JAK2 mutation. No genetic abnormalities were identified. An echocardiogram with bubble study was also obtained and showed no evidence of right to left shunting. The patient’s thrombocytosis was determined to be a reactive event that occurred due to severe hemorrhage during delivery that was superimposed on pre-existing iron-deficiency anemia from pregnancy. Follow-up CT scans of the head at 6 and 24 h were unchanged and showed no evidence of hemorrhagic transformation. The patient improved clinically and was discharged on therapeutic-dose enoxaparin and hematology/oncology follow-up after a four-day hospital stay.

## Discussion

Cervical artery dissection, including VAD and carotid artery dissection, are rare complications of pregnancy. Events most commonly occur in the postpartum period with an estimated annual incidence of 2.6–3 per 100,000 pregnancy hospitalizations [4]. VAD has been associated with HDP [2, 5]. Leffert et al. performed a national cross-sectional study of over 81 million pregnancy hospitalizations and demonstrated that women with HDP were 5.2 times more likely to have a stroke than those without HDP. Authors also noted that the national rate of HDP-associated stroke had more than doubled from 1994 to 95 to 2010–11 while rates of non-HDP-associated stroke had only increased by 47% over the same time period.

VAD is a rare cause of stroke, with an estimated annual incidence of 1 per 100,000 individuals. Events most commonly occur in young individuals, who typically present with nonspecific symptoms such as vertigo, headache, and neck pain. VAD is classically associated with trauma or connective tissue disorders. However, one recent meta-analysis demonstrated that nearly 50% of cases occur in the absence of such risk factors [6].

Aortic, cervical, and coronary artery dissection in the postpartum state have been described in the medical literature [7]. However, to our knowledge, there are only 11 previously reported cases of isolated VAD in the postpartum state (Table 1) [2–4, 8–11]. Headache or neck pain has been present in every case report of isolated postpartum VAD. In 5 of 11 patients (45%), the headache was a severe, “thunderclap” headache. 11 of 12 reported cases involved individuals over the age of 30 years, with an average age of 34 years (range: 27–41 years).

**Table 1:** Summary of cases of isolated post-partum VAD

Author	Age	Presenting Symptoms	Risk Factors	Affected Vertebral Artery	Delivery	Time from Delivery (Days)
Current report	31	Frontal headache, vertigo, nausea, vomiting	Preeclampsia, smoking	Left	Cesarean	10
Shanmugalingam et al., 2016 [2]	30	Headache, ipsilateral neck pain	NSAID-induced postpartum HTN	Right	–	6
	30	Ipsilateral neck pain	Prior IUGR and postpartum eclampsia	Left	–	6
Finley et al., 2015 [4]	35	Thunderclap headache	Migraine	Right	–	21
Nishimura et al., 2015 [8]	35	Thunderclap headache	Eclampsia, PRES	Right	–	8
Kelly et al., 2014 [9]	39	Thunderclap headache, ipsilateral neck pain, blurred vision	HTN, hyperlipidemia	Bilateral	Vaginal	21
Drazin et al., 2012 [10]	37	Thunderclap headache	–	Bilateral	–	3
Arnold et al., 2008 [3]	41	Bilateral neck pain	Migraine, hyperlipidemia	Left	Vaginal	18
	27	Ipsilateral neck pain, thunderclap headache	Migraine, HTN, hyperlipidemia	Right	Vaginal	11
	38	Thunderclap headache	Migraine, hyperlipidemia	Bilateral	Vaginal	7
	34	Ipsilateral neck pain, headache	Chiropractor neck manipulation	Right	Vaginal	7
Gasecki et al., 1999 [11]	34	Headache, neck pain		Right	Vaginal	14

A*bbreviations*: *HTN* Hypertension, *IUGR* Intrauterine growth restriction, *NSAID* Non-steroidal anti-inflammatory drug, *PRES* Posterior reversible encephalopathy syndrome

The etiology of postpartum VAD remains to be definitively established. Borelli et al. proposed a dual mechanism of pathogenesis: (1) advanced age causes increased arterial stiffness and (2) hormone fluctuations induce structural vascular changes [1]. McKinney et al. also suggested that endothelial damage may occur due to release of vasoactive or angiogenic substances during pregnancy [12]. In a retrospective review comparing postpartum versus non-postpartum VAD, Arnold et al. noted that postpartum VAD more often occurred in association with other vascular conditions, such as reversible cerebral vasoconstriction syndrome, reversible posterior leukoencephalopathy syndrome, and subarachnoid hemorrhage [13]. Maternal trauma during delivery has been excluded as a contributory factor for VAD, as the time of presentation may vary from 6 to 21 days postpartum. In addition, VAD has been reported both in mothers who delivered vaginally and via cesarean section.

The differential diagnosis for postpartum headache is extensive and includes large artery atherosclerosis, penetrating small artery disease, embolism, dissection, migraine, fibromuscular dysplasia, coagulopathy, drug abuse, and giant cell arteritis [4]. Postpartum headache and vertigo are relatively common and thus establishing a diagnosis of VAD may be challenging. Indeed, some investigators have speculated that VAD is likely underdiagnosed [5].

Our case is unique because our patient presented with an isolated left vertebral artery dissection in the setting of reactive thrombocytosis secondary to acute hemorrhage from delivery and subacute blood loss from persistent vaginal bleeding, both of which were superimposed on iron-deficiency anemia from pregnancy. Previous cases of postpartum VAD did not report on laboratory abnormalities such as anemia or thrombocytosis. Our case offers potential insight into the pathogenesis involved in postpartum VAD, given our patient’s reactive thrombocytosis. It is also conceivable that our patient’s thrombocytosis was worsened by preeclampsia, as it has been established that platelet counts may increase by two to threefold 6–14 days after a preeclamptic pregnancy [14].

Although usually benign, reactive thrombocytosis and iron-deficiency anemia have been reported as rare causes of stroke [15, 16]. A decreased hemoglobin compromises the oxygen-carrying ability of blood flow and increases the risk of cerebrovascular events [16]. Freilinger et al. reported a 43-year-old female with a 10-year smoking history and V617F JAK2 essential thrombocythemia (ET) who presented with spontaneous dissection and occlusion of the right internal carotid artery. Although speculative, the authors proposed that prothrombotic changes secondary to ET and a history of tobacco use disturbed the microcirculation within the vaso vasorum and increased the vulnerability of the vessel wall [17]. Though our patient tested negative for the V617F JAK2 mutation, our patient’s platelet count was higher than the platelet count reported by Freilinger et al. (1,003,000 /μL vs 700,000 /μL), and she similarly had a history of prior tobacco use.

We propose that a combination of our patient’s reactive thrombocytosis, tobacco use, anemia, post-partum state, and preeclampsia led to a pro-thrombotic state and weakened the vertebral artery vessel wall. Specifically, the patient’s reactive thrombocytosis played a key role in the prothrombotic state and possible disruption of the vaso vasorum leading to dissection and occlusion. The relationship between reactive thrombocytosis and cerebrovascular events is an area that requires further investigation. Clearly, diagnosing post-partum VAD requires a high index of suspicion. Fortunately, a diagnosis of VAD was established quickly, and our patient experienced a rapid and uncomplicated recovery with no lasting neurologic deficits.

## Conclusion

We present the case of a 31-year-old woman who presented 10 days postpartum with a posterior cerebellar stroke secondary to VAD, and we summarized known cases of isolated VAD in pregnancy in the literature. To our knowledge, this is the first report of isolated postpartum VAD with reactive thrombocytosis secondary to overlapping iron-deficiency anemia and acute hemorrhage. VAD is a rare cause of neurologic symptoms in the postpartum period and should be considered in the differential diagnosis for women who present with nonspecific symptoms such as headache and/or vertigo. Women aged 30 years or older and those with a history of HDP are at particularly high risk, and reactive thrombocytosis may play a potential role in this process that requires further investigation. Prompt diagnosis and management of VAD is essential to ensure favorable patient outcomes.

## Data Availability

Data sharing not applicable to this article as no datasets were generated or analysed during the current study.
